# Ideal Mode of Auditory Stimulation in Preterm Neonates in Neonatal Intensive Care Unit: A Systematic Review

**DOI:** 10.7759/cureus.34496

**Published:** 2023-02-01

**Authors:** Pallavi Palaskar, Shruti D Ramekar, Namrata Sant, Rinkle J Malani

**Affiliations:** 1 Pediatric Neurology, Mahatma Gandhi Mission (MGM) School of Physiotherapy Aurangabad, Aurangabad, IND; 2 Pediatrics, Mahatma Gandhi Mission (MGM) School of Physiotherapy Aurangabad, Aurangabad, IND; 3 Physiotherapy, Mahatma Gandhi Mission (MGM) School of Physiotherapy Aurangabad, Aurangabad, IND

**Keywords:** sensory stimulation, neonatal intensive care unit, systematic review, auditory stimulation, preterm neonates

## Abstract

The objective of this review was to find out the best mode of auditory stimulation for preterm neonates admitted to the neonatal intensive care unit. We also aimed to find out the different effects of different types of auditory stimulation in these neonates. Advanced neonatal care and technological advances in neonatal intensive care units have led to increased survival of preterm-born neonates, but this in turn leads to increased incidences of disabilities like cerebral palsy, visual impairment, delayed social development, etc. Early intervention is provided to facilitate further development and prevent delays in all domains. Auditory stimulation is proven to benefit these neonates to stabilize their vitals and improve their auditory performance in later life. Different modes of auditory stimulation have been studied worldwide, but none of the studies has presented the ideal mode of auditory stimulation for these preterm neonates. In this review, we have discussed the effects produced by different types of auditory stimulation and compared their pros and cons. For conducting a systematic review, a search strategy adopted by MEDLINE is used. A total of 78 articles published between 2012 and 2017, on the effects of auditory stimulation on preterm infants’ performance were reviewed. Out of that, eight studies that met the inclusion criteria and focused on short-term and long-term effects were included in this systematic review. Search terms included preterm neonates, auditory stimulation, and early intervention. Randomized controlled trials and cohort studies were included. Auditory stimulation by maternal sound provides physiological and autonomic stability, but the behavioral states of preterm neonates improved with auditory stimulation by music therapy with lullabies. Maternal singing during kangaroo care may be recommended for providing physiological stability.

## Introduction and background

In the last three decades, technological advancement leads to improvised NICU care for neonates which leads to reduced neonatal mortality. Survival of preterm neonates has increased and so the associated neonatal disability has also increased. preterm births hamper the development of neonatal senses, and it ultimately leads to lots of sensory issues in these neonates later. The auditory system is very important as it is responsible to receive auditory information, processing, and further speech and language development of these children. There are different protocols available for the stimulation of the auditory system and its development, but still, clear practice guidelines are not available, so this review is planned to study the different protocols of auditory stimulation and thorough guidelines may be generated for future reference for auditory stimulation of these neonates.

In the third trimester of pregnancy, the auditory system of the fetus is functional [1]. In previous studies, it was proved that the fetus shows a response in terms of changes in physiological parameters to sounds of varied acoustic frequencies. Evidence is supporting that fetuses are sensitive to sound [2-4]. The voice of the mother is the most frequent sound the fetus gets exposed to. That is the reason they respond much better to the voice of their mother than any other sound [5-9]. Evidence supports that neonates can differentiate and show a preference for the voice of their mother over any other sound [10]. Male and female voices can be differentiated by a fetus at 30 weeks of gestational age (GA) [11-14]. They can also respond to the sound directed to them [6], like speech and music [3]. Different types of speech can be learned by fetuses in the womb itself as well as they can differentiate between the sounds of different acoustic frequencies. They can also recognize the differences in the voice of mothers and other females who are strangers to them [15].

As these preterm-born neonates experience NICU stay so auditory stimuli during intrauterine age are totally different from that of NICU. Even the sensory experience of these neonates in the NICU is different from that of other preterm neonates without a NICU stay. They have less exposure to maternal voice, maternal touch, and visual input of the mother’s face. Rather, during NICU stay these neonates experience noises of medical equipment, and visual input from others' faces and more often these sensory experiences are unpredictable and involve unpleasant sensations. The NICU procedures hamper normal sensory experiences and rather these neonates have to listen to a variety of harmful and strange auditory stimuli, like noises in the neonatal intensive care unit. Evidence supports the positive effects of the voice of own mother on a neonate’s physiological stability for a short duration [16-18]. A number of studies have shown that when maternal recorded voices were given as auditory stimuli, the tolerance to feed and enteral feed was improved earlier in them as compared to the control group. They have also shown positive changes in terms of oxygen levels in the blood as well as the frequency of cardiovascular events was less reported in this group [19-21]. Most of the studies on preterm-born infants admitted in the neonatal intensive care units, with different types of auditory stimuli, such as the singing of a lullaby, the sound of instruments proved effective. A number of studies on music interventions as an auditory stimulation in the NICU have proved that preterm infants had more stable physiological parameters and reduced duration of hospital stay, improvement in weight gain at a faster rate [22-25].

In NICU, along with different kinds of sounds from medical equipment like alarms, ventilators, etc., there are noises created by talks of other family members and NICU staff. So, these auditory stimuli may hamper the neurodevelopment of these neonates. A graded auditory stimulation may definitely help these neonates for further development of the auditory system and later on may have a positive impact on the language and speech development of these neonates. Nowadays, even fathers are also involved in providing care to newborns but less studies are available to conclude the role. There are so many studies on auditory stimulation to this neonate few of them have studied the impact of auditory stimulation by the live voice of the mother or recorded mother’s voice or recorded rhymes sung by biological mothers etc. and found that mother’s voice had very much positive impact on physiological stability of these neonates. Auditory stimulation in the form of singing during kangaroo care by the mother has also been studied and it was found to be effective in providing physiological stability to these neonates. So many studies had used music therapy given by music therapists as an auditory stimulation and found that it has a positive outcome in terms of long-term speech and language development of these preterm-born NICU graduates. Most of the studies on music therapy had given auditory stimulation by singing a rhyme or a song sung by a neonatal music therapist. Few studies on music therapy had used instrumental music for neonatal auditory stimulation [20-26]. Yet none of the studies was conducted to find out the comparatively best type of auditory stimulation to be given to these neonates. Hence this systematic review is planned to study the better protocol of types of auditor stimulation to be given to these neonates for better development of the auditory system and so on the further development of speech and language of these neonates in the future.

## Review

We followed PRISMA guidelines for methodology.

Registration and protocol

The systematic review was registered with Prospero; an international prospective register of systematic reviews. CRD nois CRD42020206397.

Search strategy

Later, the papers were searched by web-based search engines. PubMed, Cochrane reviews, Biomed central, and Google scholars search databases were used. The following keywords were used for searches preterm neonates, neonatal intensive care unit, auditory development, auditory stimulation, and speech and language development. Filters were not added to searches as not much research is available on them.

Eligibility Criteria

The following selection criteria for systematic review were used.

Inclusion criteria: The following studies were included where infants with GAs less than 36 weeks of GA at birth, the studies where auditory stimulation was given in the form of recorded or live maternal voice, maternal singing, creative music therapy, singing by kin, studies included were verified for the details of the intervention as well as were checked whether the intervention was given in a NICU, the outcome of the intervention was confirmed by a specific measurement. The studies included in this systematic review were published between 2012 and 2017. The studies included were restricted to auditory stimulation of neonates in the NICU.

Exclusion criteria: Studies that included neonates with a gestation age of more than 36 weeks, studies with poor methodology, dissertations unpublished research work, etc., were not included in this systematic review.

Study selection

Four reviewers have worked out this systematic review. The reviewers had gone through detailed screening to find out whether similar data had been published previously or not. The abstracts of all articles were studied thoroughly, and the relevant articles were screened and read in detail from the full text of those articles. Relevant information was retrieved from full-text articles and all the reviewers have stringently adhered to the criteria of inclusion. The language of all articles included in this study was English and articles published till 2017 were included based on the above-mentioned selection criteria. Only the published papers were included. Dissertations and unpublished studies were not included in this review.

Data collection

The first and second reviewers had gone thorough search of all the articles related to auditory stimulation of preterm neonates in NICU and then retrieved the information from relevant articles then the third reviewer had done rechecking of all articles for accuracy and finalized them for inclusion in this study based on the selection criteria. The following data for each paper like the title of the study and author, year of publication, type of study, participants included, details of treatment given, outcome measures, and results were extracted.

Qualitative appraisal

Qualitative analysis was done during the selection of articles to be included in the study. PEDro scale was used to assess the quality of articles included in this review. Articles with poor PEDro scores were not included. All articles included in the study were checked for PICO format. Quantitative analysis was impossible due to heterogeneity in the articles included in this systematic review.

Data analysis and results

**Figure 1:**
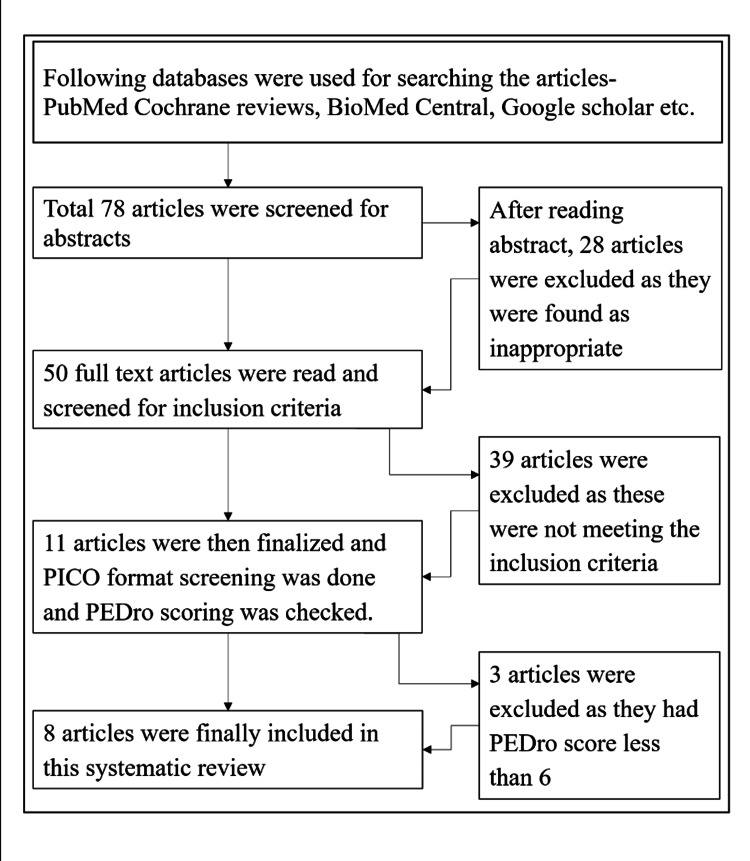
Flow diagram of search process and selection process

Eight studies based on the criteria of inclusion were included in this review. All the articles included in the systematic review were checked for PICO format. Then PEDro scores of all articles were checked and articles with PEDro scores of 6 and more were included in this systematic review to ensure the quality of the research. The detailed procedure for conducting this review was explained in Figure 1.

Then, the steps followed are explained here in an organized way. The abstracts of a total of 78 articles on auditory stimulation were screened. Then 28 articles were excluded considering their title and content. The full text of the remaining 50 articles was read and screened for inclusion criteria. Out of these 50 articles, 39 articles did not meet the inclusion criteria, so they were excluded from the study. Now, a total of 11 articles were left out of the three that had a PEDro score of less than 6, so they were excluded, and eight articles were finalized for inclusion in this review.

A total of eight studies were included and reviewed thoroughly in this systematic review. It provided evidence that hearing maternal voices by neonates admitted to NICU provides stability of vital parameters of these neonates. In the first month of life, the preterm neonates respond to the voice of the mother with a reduced heart rate. The auditory stimulation by the maternal voice of these preterm-born neonates is possibly providing autonomic stability to these neonates. It may provide a more relaxing environment to this population. The evidence of the constantly decreased heart rate of preterm neonates in response to maternal voice encourages the practices of auditory stimulation of these neonates using maternal voice for providing physiological stability. It may also help in the development of the auditory system and language. The articles included in this systematic review encourage the practice of singing lullabies by the mother while giving Kangaroo care for proving autonomic stability to these preterm-born infants. The analysis of the studies included in this systematic review showed the maternal voice, maternal singing or pacifier-activated music player with maternal voice may be useful to these preterm infants in terms of providing autonomic stability, stabilizing physiological parameters, or improving oral feeding which in turn may help them for further development. The analysis of the articles included in this study suggested that the auditory stimulation by the voice of the mother is helpful in terms of weight gain and feeding capabilities, in addition to this it may help to provide physiological and autonomic stability, but these articles did not show a significant effect on the behavioral state of these infants. Table 1 includes potentially relevant articles included in the study and the PICO information of the articles included [16-19].

**Table 1:** PICO Information of all articles included in the review

Researchers (date)	Study design	Statistical significance	Participants		Treatment description					Outcome measures	Results
			Number	GA	Type of stimuli	Delivered by	Mode of delivery	Sound level	Procedure		
Laura Doheny, Shelley Hurwitz, Robert Insoft, Steven Ringer, Amir Lahav, 2012											
Joanne Loewy, Kristen Stewart, Ann-Marie Dassler, Aimee Telsey, Peter Homel, 2013	A randomized clinical multisite trial	Statistically significant	272 premature Infants in 11 NICUs	aged >32 week	Auditory	by a certified music therapist	The informed, intentional therapeutic use of live sound and parent-preferred lullabies	55-65 dB	Infants received 3 interventions per week within a 2-week period, when data of physiologic and developmental domains were collected before, during, and after the interventions or no interventions and daily during a 2-week period	infant’s vital signs, feeding behaviors, and sleep patterns were recorded	The informed, intentional therapeutic use of live sound and parent-preferred lullabies applied by a certified music therapist can influence cardiac and respiratory function.
Olena D. Chorna, James C. Slaughter, Lulu Wang, Ann R. Stark, Nathalie L. Maitre, 2013	Randomized parallel group trial	significant	94	34-36 weeks	Auditory	Mothers voice	Pacifier activated mother voice		During the entire hospital stay, per standard of care, infants in both groups were offered pacifiers by nursing staff and mothers for NNS whenever they were TICL in a quiet-alert state before a feeding time.		A PAM using mother’s voice improves oral feeding skills in preterm infants without adverse effects on hormonal stress or growth.
Katherine Randa, Amir Lahav, 2014	Clinical trial	significant	20 hospitalized preterm infants	25 and 32 weeks of gestation	Maternal voice	Nurses	recordings		Neonates was exposed to recorded maternal sound for 30 minutes and HR changes were recorded	HR changes recorded	Preterm newborns responded to maternal sounds with decreased heart rate throughout the first month of life and improves autonomic stability of these neonates.
Shmuel Arnon, Chagit Diamant, Sofia Bauer, Rivka Regev, Gisela Sirota, Ita Litmanovitz, 2014	A prospective randomized, within-subject, crossover, repeated-measures study		86 stable preterm infants	postmenstrual age of 32– 36 weeks	Auditory	Maternal	Maternal singing during kangaroo care led to autonomic stability in preterm infants and reduced maternal anxiety	50–65 dB	Each therapy session started 30 min after completion of feeding. The sessions began with 10 min of KC therapy alone, followed by either KC alone or the intervention of KC and live Maternal singing for 20 min. The sessions ended with another 10 min of KC therapy alone. The two therapies were performed (with mother–infant dyads) over 2 days, alternating according to the randomization schedule		Maternal singing during KC reduces maternal anxiety and leads to autonomic stability in stable preterm infants. This effect is not detected in behavioral state or physiological parameters commonly used to monitor preterm infants.
Amir Lahav, Erika Skoe, 2014	Repeated measures design	NA		<36 Weeks	Auditory	NICU noise	NICU talks and equipment noise	60-70 dB	Exposure to high frequency NICU noise creates acoustic gap in preterm neonates.		High-frequency frequency noise exposure in the NICU is a concern because the auditory system is still functionally under- developed at 5birth
Friederike Barbara Haslbeck, Hans-Ulrich Bucher, Dirk Bassler, Cornelia Hagmann, 2017	Randomized controlled trial	significant	60 Stable preterm infants	<32 weeks of gestational age	Auditory	Music therapist	singing		Creative music therapy, Music therapy intervention is approximately 20 min in duration three times per week. A trained music therapist sings for the infants in lullaby style, individually entrained and adjusted to the infant’s rhythm and affect	Neurobehavioral outcomes	Results suggested that the creative musical therapy may be beneficial for neurodevelopment of child
Friederike Barbara Haslbeck, Hans-Ulrich Bucher, Dirk Bassler, Cornelia Hagmann, 2017	A prospective randomized controlled trial		60 clinically stable preterm infants	<32 weeks of gestational age	Auditory	Certified music therapist	Music therapy intervention is		Approximately 20 min in duration three times per week		

Our analysis of articles about auditory stimulation of these preterm-born infants by maternal sound has shown a correlation between the effects of maternal sound and the age of the infants. It has been found that the episodes of cardiorespiratory events were less with maternal sound in the infants with GA ≥33 Weeks. The possible explanation for this could be that the infant’s brain auditory development is intact at 33 weeks. It can be recommended that auditory stimulation of preterm neonates with maternal sound may be more beneficial at 33 weeks and above. A therapeutic implication can be suggested that maternal sound will provide more physiological stability and reduce cardiorespiratory events if started at 33 weeks of GA [19].

Apart from maternal sound, the different modes of auditory stimulation were also analyzed in this systematic review. One of them is NICU music therapy in which a recorded lullaby sung by kin is given as an auditory stimulation. A song of a kin intervention was provided to 272 neonates. In this intervention, a song that included a culturally based, personally selected music tune by parents was used. This intervention is provided by a certified music therapist. A song of a kin intervention included a song sung either by the mother or father or kin. Auditory stimulation of neonates by this way of music therapy has supported the evidence of sustained heart rate responses over time but all other physiological parameters were not showing significant differences when compared to other modes of music therapy like using Twinkle-Twinkle melody. Rather it has been found that there was slightly better oxygen saturation with the Twinkle-Twinkle melody than there was with a song of kin intervention [20].

For the therapeutic window, it can be suggested that repeatedly singing familiar songs, like the song of kin can be useful during pregnancy and it may also show good physiological and neurological influence before and after the birth. A lullaby can greatly improve the activity of an infant and quiet alert state. When a song of kin was used as an intervention, the sucking rate was significantly improved so it can be used for therapeutic purposes.

It has also been suggested that the sucking rate was profoundly active. Music therapy, in turn, when compared with maternal sound had shown positive effects on the behavioral state as maternal sound did not show significant effects on the behavioral state. It can be suggested that for inducing positive quiet alert states and behavioral maturation, music therapy may be preferred as compared to maternal sound alone.

For providing both physiological and autonomic stability and better behavioral states in terms of positive quiet alert states, music therapy by the song of a kin intervention in maternal voice may be more beneficial. In this systematic review one follows up research is also included to study the long-term benefits of auditory stimulation by Creative music therapy. This study supported the use of creative music therapy for improving the structure and functions of the brain and neurobehavioral outcomes in preterm infants [21].

## Conclusions

Auditory stimulation by maternal sound provides physiological and autonomic stability, but the behavioral states of preterm neonates improved with auditory stimulation by music therapy with lullaby. Maternal singing during kangaroo care may be recommended for providing physiological stability. However more studies in the form of RCT for comparison of effects produced by different types of auditory stimulations of neonates in NICU with different age groups are needed to draw a concrete conclusion.
